# Dynamic Computation Offloading Scheme for Drone-Based Surveillance Systems †

**DOI:** 10.3390/s18092982

**Published:** 2018-09-06

**Authors:** Bongjae Kim, Hong Min, Junyoung Heo, Jinman Jung

**Affiliations:** 1 Division of Computer Science and Engineering, Sun Moon University, Asan 31460, Korea; 2 Division of Computer and Information Engineering, Hoseo University, Asan 31499, Korea; hmin@hoseo.edu; 3 Division of Computer Engineering, Hansung University, Seoul 02876, Korea; jyheo@hansung.ac.kr; 4 Department of Information and Communication Engineering, Hannam University, Daejeon 34430, Korea

**Keywords:** drone, offloading, surveillance

## Abstract

Recently, various technologies for utilizing unmanned aerial vehicles have been studied. Drones are a kind of unmanned aerial vehicle. Drone-based mobile surveillance systems can be applied for various purposes such as object recognition or object tracking. In this paper, we propose a mobility-aware dynamic computation offloading scheme, which can be used for tracking and recognizing a moving object on the drone. The purpose of the proposed scheme is to reduce the time required for recognizing and tracking a moving target object. Reducing recognition and tracking time is a very important issue because it is a very time critical job. Our dynamic computation offloading scheme considers both the dwell time of the moving target object and the network failure rate to estimate the response time accurately. Based on the simulation results, our dynamic computation offloading scheme can reduce the response time required for tracking the moving target object efficiently.

## 1. Introduction

Various research works related to surveillance and object tracking have been performed continuously [1,2,3,4]. These systems are evolving from a surveillance system using fixed monitoring devices to a surveillance system using mobile monitoring devices. In the case of a mobile surveillance system, there is the advantage that the distance that can be monitored is not limited because it is not fixed when compared to a typical surveillance system. Therefore, mobile surveillance systems can be used for recognizing and tracking suspicious persons or objects. When making a mobile surveillance system, it is very attractive to use drones to cover a wider range.

**Figure 1:**
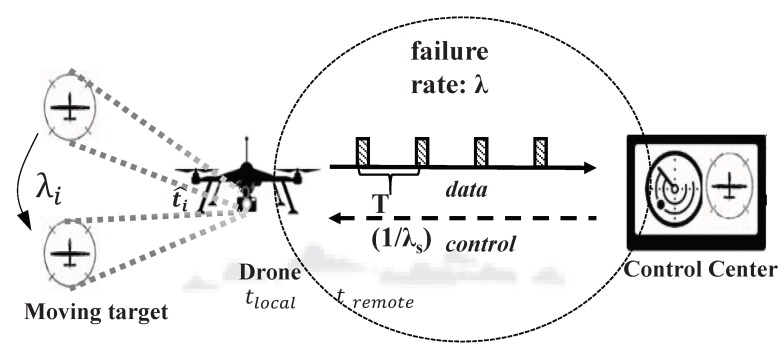
A concept for a drone-based mobile surveillance system.

Figure 1 shows a concept for a drone-based surveillance system. Generally, as shown in Figure 1, the drone-based mobile surveillance system can consist of three major parts: a moving target, a drone for tracking and a remote control center for controlling the drone. The drones can recognize or track moving target objects using the attached camera. The remote control center can control the movement of the drone or monitor the status of the drone.

As we explained before, drone-based mobile surveillance systems have some advantages when compared to the previous typically robot-based mobile surveillance systems. Typically, drone-based mobile surveillance systems have a larger surveillance coverage than previous robot-based mobile surveillance systems [3]. However, robots or drones used for constructing mobile surveillance systems operate on a limited battery. In addition, the computing resources of the drone may not be sufficient to track or recognize moving objects within some time constraints such as total execution time, response time and deadlines.

The remote control center can fulfill some operations or jobs related to object tracking instead of the drone. The control center can return the tracking results back to the drone by using wireless communication technologies after performing the operations or jobs. Therefore, it is not reasonable to perform all computations related to object tracking or recognizing by the drone in terms of the response time or the energy consumption of tracking. Dynamic offloading schemes can be used to overcome these problems.

In our previous work [5], we proposed a conceptual model, which is a dynamic computation offloading policy for drone computation. In this paper, we extend the previous research and propose a dynamic computation offloading algorithm for drone-based mobile surveillance systems. In our scheme, we consider several factors such as the mobility of the moving target object, computation or operating delay time on the drone and network link failure rate between the drone and the remote control center. Our scheme can dynamically offload computation operations or jobs related to tracking or recognizing objects while considering both the expected dwell time of the moving object and the network link failure rate. Additional energy consumption may occur to determine whether to offload or not. In this paper, we focused on the analysis to reduce the response time for object tracking. We performed simulations to evaluate and analyze the performance of the proposed dynamic computation offloading scheme in terms of the response time. Based on the simulation results, our dynamic computation offloading scheme can reduce response time for object tracking.

The rest of the organization of this paper is as follows. In Section 2, we describe some related works. We will explain our dynamic offloading algorithms for drone computation in Section 3. As we explained before, our dynamic offloading scheme considers the dwell time of the target object and the link failure rate between the drone and the remote control center. In Section 4, we will evaluate and analyze the performance of the proposed dynamic offloading algorithms in terms of the response time. In Section 5, we conclude this paper.

## 2. Related Works

There are many studies related to mobile video surveillance systems that transmit collected data to the destination under tight time constraints such as delay, accuracy and mobility [2]. In these systems, a mobile monitor traces an active object with its tracking devices such as various sensors and camera. Collected data are used for recognizing the active object and controlling the next position of the monitor and its tracking devices to continue to track the object. In this section, we explain the computation offloading of the collected data and the controlling schemes of the Pan-Tilt-Zoom (PTZ) camera, respectively.

### 2.1. Computation Offloading

Computation offloading is widely used to overcome the computational power limitations of a resource-constrained mobile monitor [6]. Offloading performs some parts of a task or a job of the mobile monitor on servers on behalf of the mobile monitor. If the execution cost of the computation operations in the part of the task in the mobile monitor is greater than the execution cost considering the offloading mechanism, the part of the task is performed on remote servers. In some applications such as mobile video surveillance systems, the offloading decision scheme considers real-time features, as well as energy efficiency. Nimmagadda et al. [3] proposed a real-time moving object tracking scheme using mobile robots based on computation offloading. Their offloading decision scheme estimates the computation and communication time of the offloaded task and chooses to execute the task on a robot or servers to satisfy time constraints and minimize the total response time.

In [7], the authors proposed a surveillance system named MOCHA (Mobile Cloud Hybrid Architecture) for a face recognition system that can be used for airport security and surveillance. MOCHA was devised to reduce the overall processing time for face recognition. One of the important issues is how to divide computation loads among the servers in order to get the minimal response time given different network delays and server computing capabilities. In the proposed system, mobile devices capture images and send the captured images to the cloudlet. After that, the cloudlet fulfills image processing computations on the received image data and finds matching images from the database.

In [4], the authors devised scheduling algorithms that reserve server resources to meet the deadline for offloaded tasks for real-time systems. the authors considered frame-based real-time tasks. In addition, all the tasks have the same arrival time, relative deadline and execution period. In this circumstance, the scheduler manages the real-time tasks on the client side. In addition, the scheduler determines which tasks to be offloaded from the local to the remote server in order to minimize the required utilization and meet the required time constraints.

Chen et al. [1] proposed a continuous, real-time object recognition system for camera-equipped mobile devices called Glimpse. Glimpse captures video, recognizes and labels objects of interest and tracks them frame to frame. It fully offloads these tasks to servers because the algorithms for object recognition and labeling include significant computation overhead. It also adjusts the recognition accuracy adaptively with the network latency.

### 2.2. Tracking an Active Object with a PTZ Camera

A Pan-Tilt-Zoom (PTZ) camera can be remotely controlled and trace a moving object by adjusting its position. The PTZ camera also conducts one of the important roles in visual surveillance systems. Many studies have been conducted related to tracking an active object with the PTZ camera. In [8,9], the authors used a PTZ camera for face tracking and recognition. The former is composed of a coaxial-concentric camera system with a PTZ camera, assisted static cameras and a beam splitter to improve the accuracy of the face tracking and recognition system. They designed a linear prediction model. In addition, they proposed a method to control the PTZ motion velocity for robust tracking. This system captures high-resolution face images within 12 m. The latter uses the zoomed-out and -in mode to extract a set of face image data from a scene. The extracted face information is correlated with the corresponding people and their trajectories. This visual tracker is periodically updated based on sample collection and appearance learning that classify positive samples and negative ones and extract common features among positive samples.

In the active object tracking systems, it is important to find out a target object in a captured image. Dong et al. [10] proposed an automatic tracking and detection algorithm for detecting an active object. In their algorithm, the Gaussian mixture model is used to recognize an active object. The fusion algorithm of the Kalman filter and Camshift algorithms is applied to track the active object. In [11], they proposed a PTZ tracking method that adaptively combines the appearance correlation filter response and motion likelihood map that collects the motion features of a target object to solve collision and appearance change problems.

As we explained before, a PTZ can be remotely adjusted by an operator and also control itself automatically. In [12], the authors proposed an automatic PTZ camera control scheme based on object detection and scene partition. The object is detected by using GMM (Gaussian Mixture Model), and a PTZ camera is controlled by using a scene partition technique that divides a monitoring region into several vertical and horizontal directions. Ahmed et al. [13] proposed an active camera tracking system that is composed of a pan-tilt camera control algorithm, a visual tracking algorithm and digital video stabilization modules. The PTZ camera is controlled by using a predictive open-loop car-following control algorithm that measures the expected velocity of an active object by using a Kalman filter and smoothly changes the velocity of the PTZ.

## 3. Mobility-Aware Dynamic Computation Offloading Decision Scheme

### 3.1. Drone Computation Offloading Model for Tracking and Recognizing Moving Objects

In this section, we first introduce and explain our computation offloading model of a drone-based mobile surveillance system to recognize and track a moving object. Some assumptions are also explained. We assume that a drone is equipped with a PTZ (Pan-Tilt-Zoom) camera and can take pictures of moving objects. In the tracking and recognition process of moving objects, it is assumed that the drone should handle computation-intensive tasks. It is also assumed that such computational operations can be handled by the drone themselves or remotely by the remote control center. It is assumed that the processing capability of the remote control center is superior to that of the drone.

**Figure 2:**
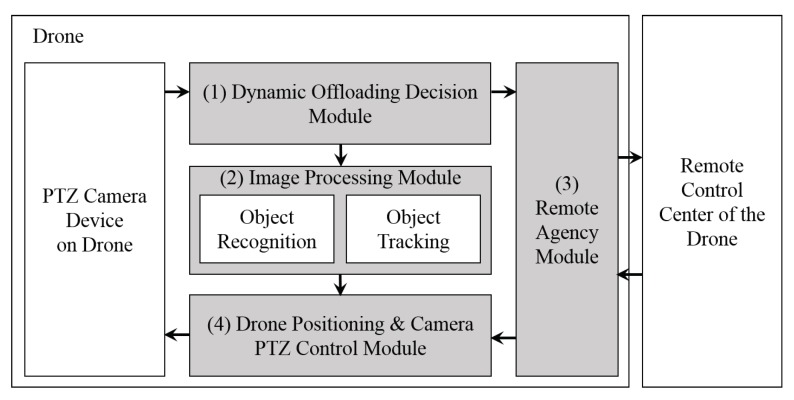
Block diagram of our drone-based mobile surveillance system.

Figure 2 shows the block diagram of our drone-based mobile surveillance system. As shown in Figure 2, a drone can recognize and track a moving object, and a part of the job can be performed in the remote control center. Our drone-based mobile surveillance system is composed of four main modules. They are a dynamic offloading decision module, a module for image processing locally, a remote agency module to communicate with the remote control center and the drone positioning and PTZ camera control module.

The dynamic computation offloading decision module is responsible for deciding whether the computations required for recognizing and tracking a moving object should be offloaded or not. When making a decision, the dynamic computation offloading decision module considers the estimated mobility information of the moving target object and network link conditions such as the network failure rate.

When the dynamic computation offloading decision module determines to perform the tasks on the drone itself, the total response time is caused by two parts, local computation time and device delay time. Let $t_{i}^{local}$ denote the execution time if the computation operations required to track and recognize the moving object are executed on the drone locally. We can divide $t_{i}^{local}$ into two parts. The first part is a local computation time $\tau_{\alpha }$, which is consumed by the CPU to execute the operations on the drone. The second part is a device delay $\tau_{\gamma }$ for the drone or its camera to move to a new position and respond to commands. We consider that $t_{i}^{local}$ is approximately constant at any time because it may be defined in the specifications of the manufacturer.

(1) $$t_{i}^{local}=\tau_{\alpha }+\tau_{\gamma }$$

(2) $$t_{i}^{remote}=\tau_{\beta }+\tau_{\gamma }$$

Conversely, if the dynamic offloading decision module decides to offload the computation operations to the remote control center, the remote agency module transfers the data required to the remote control center. Then, the remote agency gets the result of the offloaded operations from the remote control center back. Therefore, the total execution time or response time of offloaded computation operations is specified as the total amount of time required to respond to the drone’s offloading request by running the server codes for tracking and recognizing the moving target object at the remote control center. Thus, similar to local processing on the drone, the total response time of offloaded operation is composed of two parts. The first part is the response time $\tau_{\beta }$ with network delays caused by the network link failures. Another part is the drone device delay $\tau_{\gamma }$ that is the consumed time for moving the drone to a new position and to respond to commands by the drone positioning and PTZ camera control module.

### 3.2. Dynamic Computation Offloading Decision Considering the Mobility of a Moving Target Object

In this section, we explain how to measure the expected dwell time of a moving object. Based on the expected dwell time, we will describe the procedure of the dynamic computation offloading decision. We use a moving object tracking scheme proposed for the Glimpse system [1]. In Glimpse, the image processing module extracts feature points of the moving object in frame *n*. The image processing module estimates where those feature points could be located in frame n+k. Then, the velocity of the moving target object between frames $v_{i}$ at time *i* can be defined as follows.

(3) $$v_{i}=\frac{s_{i}-s_{i-1}}{t_{i}-t_{i-1}}\;assuming\;s_{i-1}\approx 0$$

**Figure 3:**
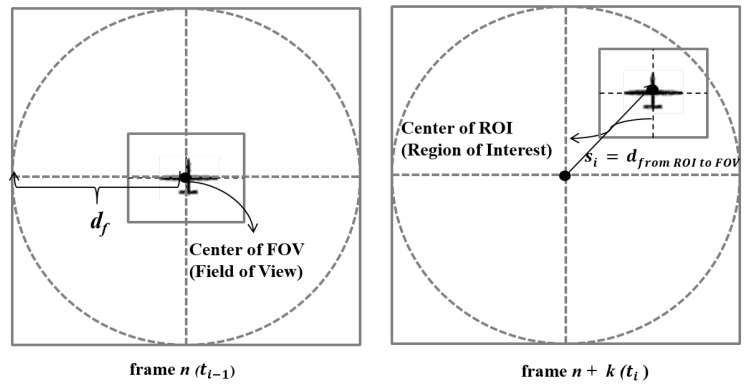
An example of the movement of a moving target object between two frames.

Figure 3 shows an example of the movement of a moving target object between two frames. The expected dwell time of a moving target object is related to the velocity of the moving object. A low dwell time of the moving target object means that the moving target object goes out of the ROI (Region of Interest) area easily in the image scene because of its quick movement. On the contrary, if the dwell time of the moving target object is high, the moving target object is likely to stay in the ROI area continuously. We can assume $s_{i-1}$ is nearly zero because the moving target object can be aligned in the center at time i−1 by using the PTZ camera of the drone. Therefore, the expected dwell time of the moving target object at time *i* can be defined by:

(4) $${\hat{t}}_{i}=\frac{d_{f}-s_{i}}{v_{i}}\;for\;d_{f}\geq s_{i}$$

where $d_{f}$ is the size of FOV (Field of View).

The expected dwell time of a moving target object ${\hat{t}}_{i}$ denotes the time that the moving target object will continuously be located in FOV (Field of View) after a time *i*. This time value can be used for an upper time constraint value for tracking or recognizing the moving object. In order to recognize and track the moving target object successfully, either $t_{i}^{local}$ or $t_{i}^{remote}$ must be lower than ${\hat{t}}_{i}$ of the moving target object.

The dynamic computation offloading decision module can determine whether to offload the job or not based on the following policies. In the cases of ${\hat{t}}_{i}$ < $t_{i}^{local}$ < $t_{i}^{remote}$ or ${\hat{t}}_{i}$ < $t_{i}^{remote}$ < $t_{i}^{local}$, successful tracking of the moving target object is impossible. In the cases of $t_{i}^{remote}$ < ${\hat{t}}_{i}$ < $t_{i}^{local}$ or $t_{i}^{remote}$ < $t_{i}^{local}$ < ${\hat{t}}_{i}$, it is much more effective to offload recognition- and tracking-related operations and process them on the server of the remote control center because $t_{i}^{remote}$ < $t_{i}^{local}$. In the cases of $t_{i}^{local}$ < ${\hat{t}}_{i}$ < $t_{i}^{remote}$ or $t_{i}^{local}$ < $t_{i}^{remote}$ < ${\hat{t}}_{i}$, on the other hand, it is more reasonable to handle them on the drone locally.

### 3.3. Considering Network Delays in Decision Making

In this section, we analyze the expected response time of offloaded computation operations on the server of the remote control center. Offloading techniques can generally be effective in reducing execution time or response time. However, offloading has a significant problem with an unexpected network delay caused by drone mobility and dynamic network conditions. Let $\lambda_{i}$ denote the network error rate at time *i*, which follows the Poisson distribution and τ be the response time without any failure of the network from the drone to the server.

If a task is performed without any failure on the server at the remote control center, the expected response time E[τ] is identical to τ. However, if a network failure occurs, we continue to retry periodically after waiting for the recovery time *r*. In this case, E[τ] can be given by three distinct terms: *x*,*r* and E[τ] recursively, where *x* is the interval between the point at which the task was initiated and the point at which the failure occurred.

(5) $$\tau_{\beta }=E\left[\tau \right]=\left\{\begin{array}{cc} x+r+E[\tau ] & \mathrm{if}x<\tau \\ \tau & \mathrm{otherwise} \end{array}\right.$$

By the law of total expectation,

(6) $$E\left[\tau \right]=\int_{0}^{\tau }\left(x+r+E\left[\tau \right]\right)f_{X}\left(x\right)dx+\int_{\tau }^{\infty }\tau f_{X}\left(x\right)dx$$

(7) $$E\left[\tau \right]=\frac{\int_{0}^{\tau }\left(x+r-\tau \right)f_{X}\left(x\right)dx+\tau }{\left\{1-\int_{0}^{\tau }f_{X}\left(x\right)dx\right\}}$$

By substituting $\lambda_{i}e^{-\lambda_{i}x}$ for $f_{X}\left(x\right)$, where $\lambda_{i}>0$, we obtain,

(8) $$\begin{array}{cc} E[\tau ] & =\frac{\int_{0}^{\tau }\left(x+r-\tau \right)\lambda_{i}e^{-\lambda_{i}x}dx+\tau }{1-\lambda_{i}\int_{0}^{\tau }e^{-\lambda_{i}x}dx}\\ & =\frac{\int_{0}^{\tau }\left(x+r-\tau \right)\lambda_{i}e^{-\lambda_{i}x}dx+\tau }{e^{-\lambda_{i}\tau x}}\\ & =\frac{\left(e^{\lambda_{i}\tau }-1\right)\left(\lambda_{i}r+1\right)}{\lambda_{i}} \end{array}$$

The remote agency module of our system is devised to provide feedback information related to the network link condition such as the network link failure rate. By using this information, the remote agency module of the drone can estimate the expected response time when offloading the computation operations. Table 1 shows the notations used in this paper.

## 4. Performance Evaluation

In this performance evaluation section, we analyze and evaluate the performance of the proposed dynamic computation offloading scheme by using simulation. Table 2 shows the parameters and the values used in the simulation. We evaluated and analyzed the performance of our proposed dynamic computation offloading scheme by comparing with local-only processing and remote-only processing methods. To the best of our knowledge, this paper is the first to investigate a dynamic offloading scheme for drones by considering network failures. For this reason, the proposed dynamic computation offloading scheme is compared with local-only and remote-only schemes. In the case of the local-only scheme, all computations for object tracking are performed locally on the drone. On the other hand, all computations for object tracking are fulfilled remotely by the server of the remote control center with more powerful computing power. Our proposed scheme can dynamically offload some computations considering the mobility information and network conditions.

**Figure 4:**
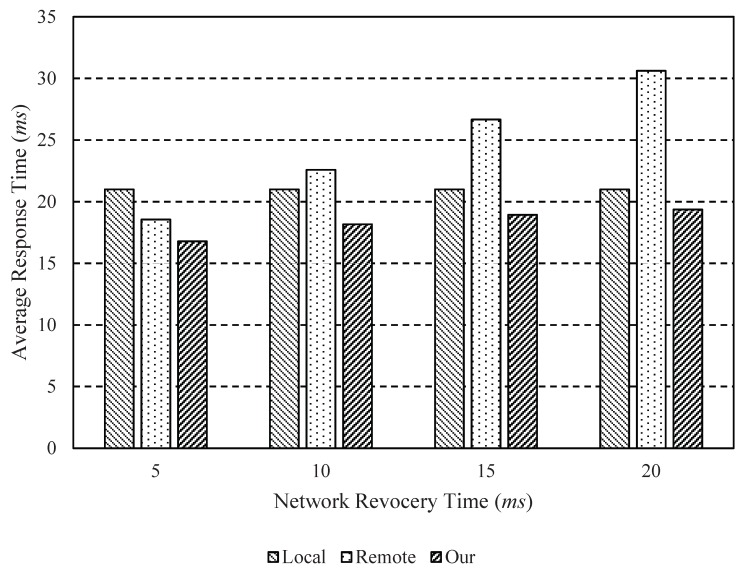
Average response time according to the network recovery time (*r*) where τ = 5 ms.

Figure 4 shows the average response time according to the network recovery time. As shown in Figure 4, our dynamic computation offloading scheme shows good performance when compared with two schemes: local-only and remote-only. The remote-only scheme shows the worst performance when compared with all schemes as the network recovery time increases. This is because the remote-only scheme offloads the job without taking into account the latency due to network link instability.

**Figure 5:**
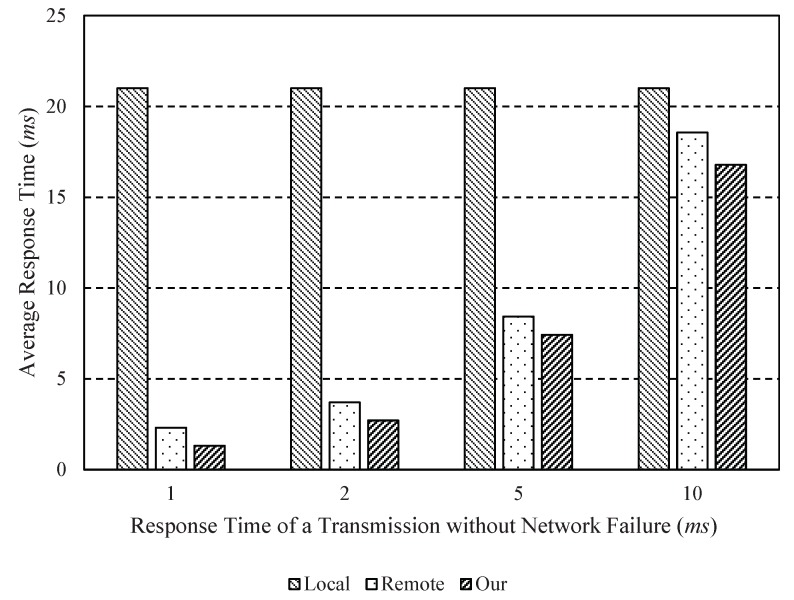
Average response time according to the response time of a transmission without network failure (τ) where *r* = 5 ms.

Figure 5 shows the average response time according to the response time of a transmission without network failure where *r* = 5 ms. As shown in Figure 5, the average response times of the remote-only and our scheme are less than that of the local-only scheme. Our scheme can reduce the average response time up to about 43.2% when compared to the remote-only scheme. In the case of the local-only scheme, there is no change in average response time because the local-only scheme performs all operations locally on the drone. Based on the above experimental results, we confirmed that the proposed method can reduce the response time required for tracking effectively.

## 5. Conclusions

Drones can be used for various purposes such as object recognition and object tracking as mobile surveillance systems. Generally, most drones have very limited battery power. Generally, in addition, the computing power of a remote control center is greater than that of a drone. Therefore, it is not good to perform all operations or computations related to recognizing and tracking of a moving target object on the drone. In this paper, we proposed a dynamic computation offloading scheme for drone-based mobile surveillance systems. The proposed dynamic computation offloading scheme can dynamically offload operations related to recognizing and tracking of a moving object to the remote control center considering the mobility information of the moving target object and network link conditions between the drone and the remote control center. Based on the simulation results, our proposed scheme showed that the average response time can be reduced effectively.

## Figures and Tables

**Table 1:** Notations used in this paper.

Notation	Description
${\hat{t}}_{i}$	The expected dwell time means the estimated time a moving target object will continuously be located in FOV after *i*
$t_{local}$	The computation time entirely on the drone
$t_{remote}$	The time when the entire computation is offloaded to a remote server
$\lambda_{i}$	The network error rate at time *i*
$\tau_{\alpha }$	The computation time entirely on drone processors
$\tau_{\beta }$	The expected response time considering network error rate $\lambda_{i}$
$\tau_{\gamma }$	The delay of the camera in executing commands
$v_{i}$	The velocity of target moving object at time
$s_{i}$	The position of the center of ROI at time *i* (square with dashed lines in Figure 1)
$d_{f}$	The FOV radius
*X*	The random variable of time at which the failure of a link occurs due to the network error
$f_{X}\left(x\right)$	The probability density function of X
*r*	The network recovery time
τ	The response time of a transmission without network failure
E[τ]	The expected response time while considering the network failure

**Table 2:** Parameters and the values used in the simulation.

Parameter	Description	Value
$\lambda_{i}$	The network error rate at time *i*	[0.01∼0.1]
$\tau_{\alpha }$	The computation time entirely on drone processors	20 ms
$\tau_{\gamma }$	The delay of the camera in executing commands	1 ms
*r*	The network recovery time	[5, 10, 15, 20] ms
τ	The response time of a transmission without network failure	[1, 2, 5, 10] ms
Simulation Time	Total execution time for the simulation	10,000 s
